# Naturally occurring and stress induced tubular structures from mammalian cells, a survival mechanism

**DOI:** 10.1186/1471-2121-8-36

**Published:** 2007-08-16

**Authors:** Yonnie Wu, Richard C Laughlin, David C Henry, Darryl E Krueger, JoAn S Hudson, Cheng-Yi Kuan, Jian He, Jason Reppert, Jeffrey P Tomkins

**Affiliations:** 1 Department of Genetics and Biochemistry, Clemson University, Clemson, South Carolina, 29634, USA; 2 Department of Biological Science, Clemson University, Clemson, South Carolina, 29634, USA; 3 Electron Microscopy Facility, Clemson University, Clemson, South Carolina, 29634, USA; 4 Department of Biosystems Engineering, Clemson University, Clemson, South Carolina, 29634, USA; 5 Department of Physics and Astronomy, Clemson University, Clemson, South Carolina, 29634, USA

## Abstract

**Background:**

Tubular shaped mammalian cells in response to dehydration have not been previously reported. This may be due to the invisibility of these cells in aqueous solution, and because sugars and salts added to the cell culture for manipulation of the osmotic conditions inhibit transformation of normal cells into tubular shaped structures.

**Results:**

We report the transformation of normal spherical mammalian cells into tubular shaped structures in response to stress. We have termed these transformed structures 'straw cells' which we have associated with a variety of human tissue types, including fresh, post mortem and frozen lung, liver, skin, and heart. We have also documented the presence of straw cells in bovine brain and prostate tissues of mice. The number of straw cells in heart, lung tissues, and collapsed straw cells in urine increases with the age of the mammal. Straw cells were also reproduced *in vitro *from human cancer cells (THP1, CACO2, and MCF7) and mouse stem cells (D1 and adipose D1) by dehydrating cultured cells. The tubular center of the straw cells is much smaller than the original cell; houses condensed organelles and have filamentous extensions that are covered with microscopic hair-like structures and circular openings. When rehydrated, the filaments uptake water rapidly. The straw cell walls, have a range of 120 nm to 200 nm and are composed of sulfated-glucose polymers and glycosylated acidic proteins. The transformation from normal cell to straw cells takes 5 to 8 hr in open-air. This process is characterized by an increase in metabolic activity. When rehydrated, the straw cells regain their normal spherical shape and begin to divide in 10 to 15 days. Like various types of microbial spores, straw cells are resistant to harsh environmental conditions such as UV-C radiation.

**Conclusion:**

Straw cells are specialized cellular structures and not artifacts from spontaneous polymerization, which are generated in response to stress conditions, like dehydration. The disintegrative, mobile, disruptive and ubiquitous nature of straw cells makes this a possible physiological process that may be involved in human health, longevity, and various types of diseases such as cancer.

## Background

Over millions of years of evolutionary time, living creatures from primitive cells to multi-cellular organisms must have been subjected to frequent episodes of dehydration; for example, bdelloid rotifers (aquatic microinvertebrates) adapt to desiccation by contracting into a compact shape and staying dormant until conditions improve [1,2]; nematodes accumulate trehalose, a non-reducing sugar, to protect membranes and proteins from desiccation [3]; plants express late embryogenesis abundant (LEA) proteins in maturing seeds and pollen in response to desiccation [4]. The adaptability of organisms in response to dehydration through a state of suspended metabolism is essential for long-term survival [2]. Dehydration may be a selective force in evolution.

Here, we report for the first time, the identification of cells that form tubular structures in response to stress. These transformed cells have been termed straw cells based on their overall tubular morphology and ability to re-hydrate and form normal cells again. Although the desiccation of mammalian cells has been included in research by various groups in studies on the mechanisms of cellular response to low moisture [5-8], tubular forms of mammalian cell response to dehydration have not been reported. This lack of detection may be due to the invisibility of the straw cells in aqueous solution, and because sugars and salts added to cell culture for manipulation of the osmotic environment inhibit transformation to a tubular form.

## Results

### Existence of straw cells in tissues and in cultures

**Figure 1:**
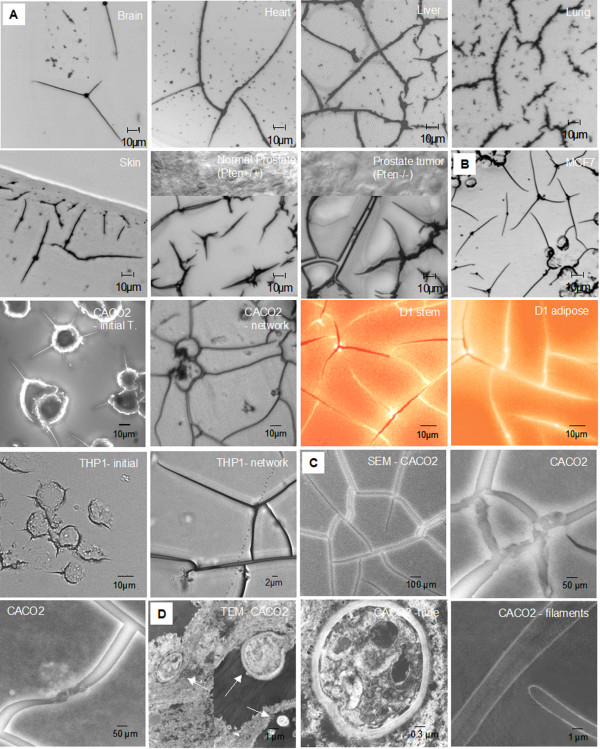
Tubular structures found in tissues and cultures. **A**. Pre-existence of tubular structures in six tissue types from fresh prepared three-month-old female calf and in frozen prostate tissues from mice. Straw cells typically possess several filament branches that connect to each other. Large quantities of straw cells were seen in liver, lung and prostate tissues followed by skin, with the smallest quantities in brain and heart tissues. **B**. Dehydration produced straw cells from five types of mammalian cells in cultures. **C**. SEM micrographs of the tubular network at 50 and 100 μm scale. Multiple straw cells connect to each other; two incoming filaments with growth tips have dark contrast, transformed cell resides inside the tube. **D**. TEM micrograph of the cross-sections of transformed straw cells. First graph shows multiple straw cells at various diameters, second graph shows a zoom-in image of the cross-section of a tube, tubular wall has a thickness 220 nm. Last graph is the cross-section of longitudinal view of filaments with diameter 1 μm, the thickness of filamentous wall is 120 nm.

A wide range of tissue types and cell lines from different mammals have been surveyed for the existence of straw cells. Freshly harvested tissues of brain, heart, liver, lung and skin of young cows, and frozen tissues of normal and tumor sections of mouse prostate have straw cells in their native environments (Figure 1A). Tubular structures in the hundreds were identified from 100 mg of brain and heart tissues, and in the tens of thousands from liver, lung and skin tissues. Approximately 800 straw cells/μl of tissue wash fluid (100 mg tissue: 100 μl water) were quantified from bovine lung tissues (freshly slaughtered at 1 to 2 years age). In experiments with cultured cells, Human MCF7, CACO2, THP-1 and mouse D1 cells formed similar tubular shapes when dehydrated (Figure 1B). Initially, very fine needle-like structures that we have termed filaments protrude from the cell surface. These filaments continue to elongate and fuse with filaments from other dehydrated cells forming an integrated network. The cell body itself becomes much smaller and assumes a tube-like structure. Cells that are detachable and mobile can cooperate in a coordinated manner by forming collaborative networks to acquire and distribute water may have increased their chances for survival.

The timing of transformation varies *in vitro*, with the majority of structures becoming visible at complete dehydration. Typically, more than 80% of cells transform with a population of 10,000 cells in 0.5 ml medium per well. Cell density, salts, the volume of the medium and drying rate affect the amount of transformation and filamentous growth. Cells dehydrated in a 2 × volume cell free phosphate saline medium and subjected to the same dehydration conditions produce no tubular structures. This suggests a nutritional requirement for negating transformation. Because the transformation mechanism appears to be conserved across mammalian cell types to include differentiated macrophage (THP-1) and adipose cells (D1), it is reasonable to consider that dehydration exhibited a selective pressure on primitive cells. Cells that are detachable and mobile can cooperate in a coordinated manner by forming collaborative networks to acquire and distribute water may have increased their chances for survival.

SEM images of straw cells and filamentous networks in solution are shown in Figure 1C. The filaments are spread out and connected to form networks, they originate from the tubular center, and the joints of the connections appear to be smooth and seamless. Cross-sections of the straw cells are shown in TEM images (Figure 1D). The samples were prepared from dehydration-transformed cells that were precipitated at 16,000 *g*, depending on the length of filamentous extensions; straw cells were either in the pellet or in the supernatant. The central tubular wall has a thickness of 200 nm; thirty times thicker than the lipid bilayer of a normal cell membrane. The wall structure appears dense and compact, and stained lightly with regular 1% osmium oxide fixation. The filamentous extension's wall is approximately 120 nm thick, 80 nm thinner than the central tubular wall. Unlike the tubular center, the filamentous extensions have a nearly perfect homogeneous content and no recognizable organelles.

The volume of a tube is much smaller than that of the main cell body, indicating that most of the cellular material has been condensed. Filaments, typically between 1 and 10 μm in diameter, often extend far from the location of the main cell body. In one instance, we observed filament growth to lengths of over 4 cm, an increase in approximately two thousand fold of the original length of the cell and seemingly confined only by the culturing well. Filaments were extended in multiple directions from cells along the culture flask as well as through the air. TEM images show the straw cell wall to be markedly robust, which probably indicates a state of dormancy and prevention of water loss.

### Straw cell properties

**Figure 2:**
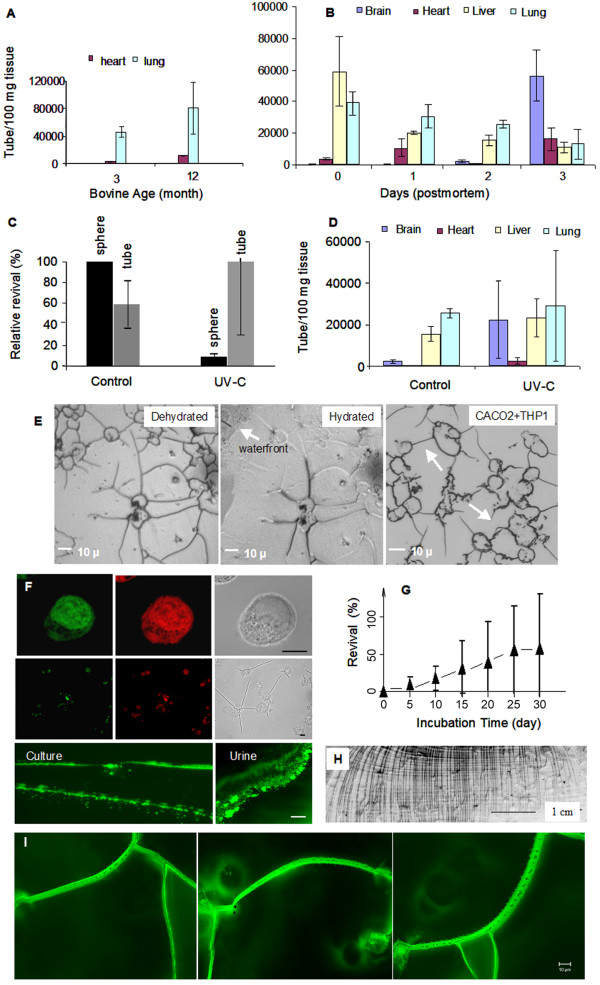
Functional assays.**A**. The population of transformed tubular structures correlates increasingly with the age of the animal. **B**. Production of straw cells in postmortem tissues.**C. **Effect of UV-C radiation on regular and transformed cells. A lethal UV-C dose to normal (vegetative) cells appears not to impair the revival of straw cells. **D**. Effect of UV-C radiation on post mortem tissues. Lethal doses of UV-C radiation do not affect the production of straw cells from post mortem tissues.**E**. First, freshly dehydrated CACO-2 cells with filamentous network on the bottom of a 4-channel Lab-Tek^® ^chamber; second, partial rehydration of tubular network with application of 1 μL water. Water rapidly moves through the network in straw cells with 1 μm diameter. Arrow points to the waterfront; third, co-dehydration of THP-1 and CACO-2 cells, filaments from THP-1 and CACO-2 are connected (arrow). **F**. Actin, nucleic acid and antibody staining of transformed CACO-2 cells. Top, normal CACO-2 cells after incubation in aqueous medium stained for actin (red) and nucleic acids (green). Initial formation of filamentous structures from cells after brief dehydration at 4 h resulted in 50% water loss (middle graph). scale bar = 10 μm. Antibody staining with rabbit anti-tube polyclonal IgG on collapsed filaments in culture and on water-soluble polysaccharides in urine (middle graph). **G**. Revival (%) of tubular structure to regular sphere shaped cells over 30 days. Straw cells remain with the supernatant at centrifugation force 16,000 *g*, and are thus separated from un-transformed cells; when re-plated in a fresh well, most regular spherical cells emerge and attach to the supporting matrix in 5 to 15 days. **H**. Purified filaments displayed on glass slide are from 1 to 4 cm in length and remain connected to each other. **I**. Fluorescent images of fixed straw cells with filamentous extensions.

Time course studies from four young animals revealed that straw cell population increases with the age of the animal in heart and lung tissues, but stays constant for brain, liver, and skin tissues (Figure 2A). When tissues *post mortem *were dehydrated in air, the straw cell population increased in brain and heart tissues. Brain tissues, when dehydrated for three days, produced straw cells comparable in number to those of liver and lung tissues at the initial stage of transformation (Figure 2B). On average the transformation from normal cells to a tubular structure occurs over an 8 h period.

A lethal dose of UV-C radiation (10 min) did not affect tubular transformation of cultured cells (Figure 2C) or in postmortem tissues (Figure 2D). UV-C radiation also seems to have no effect on straw cell reversion to a normal round state (explained in 2G). During hydration, water passes rapidly through the straw cell network. Water movement through the straw cell network was estimated on a sub-second scale (Figure 2E, the first two). Tubular structures from different cell types were connected to each other (Figure 2E, last).

**Figure 3:**
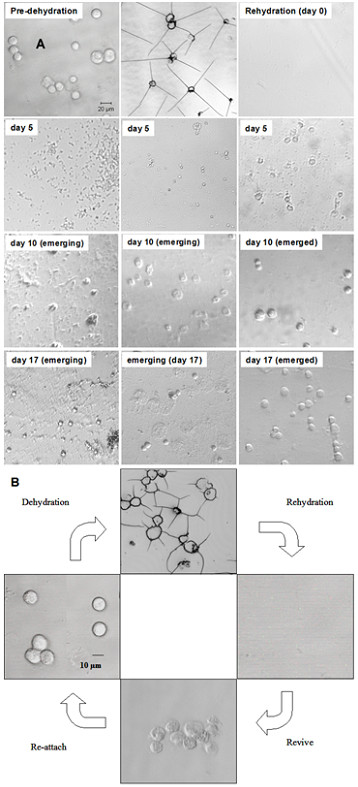
The morphology of reviving tubular structures. **A**. Time course images of the tubular structure into regular shaped cells over a period of 0 to 20 days. **B**. The life cycle of dehydration-induced straw cells. Newly emerged cell has granular surface with smaller volume and longer replication time than that of vegetative cells.

Normal cells stained positively for actin and nucleic acids (Figure 2F, first two), whereas early tubular cell bodies were barely stainable and negative for mature straw cells and filaments (the middle panels). The tubular wall appeared to be impermeable to small molecules such as ethanol, paraformaldehyde and TritonX-100 used in the staining procedure. When rehydrated, filaments quickly disintegrate. Figure 2F, left bottom, displays collapsed filaments stained with rabbit straw cell polyclonal antibody. Collapsed filaments in solution produced numerous round shaped dots similar in size to large as *E. coli *cells. Antibodies generated against purified straw cells have produced positive signals from human urine but not feces (Figure 2F, right bottom). Transformed cells obtained from non-transformed cells in vitro were re-plated in fresh medium in new 4-chambered-wells. More than 50% of the straw cells regained their normal spherical shape and began to divide in 5 – 15 days (Figure 2G). Newly recovered cells were still resistant to staining indicating that they were still in a stressed state. The morphologies of these recovering structures are presented in Figure 3.

Purified filaments were isolated from media and separated from lipid and protein fractions. The filaments were laid down on a glass slide for viewing with some strands being measured up to 4 cm in length (Figure 2H). Morphologically, the surface of the filaments is characterized by numerous hair-like structures and small circular openings (Figure 2I). Interestingly, the structures are somewhat similar to that of hairy roots from a plant whose purpose is to absorb water and nutrients.

The cycle of dehydration and rehydration is characterized in Figure 3. When rehydrated, straw cells became visible and attached to the plate bottom in 5 days. There are dark, irregular shaped bodies (day 5 and day 10 left panels) that give rise to round-shaped cells in 5 to 10 days with different morphology. Dot-shaped particles with a dimension of 1 μm (Figure 3A, day 5, middle panel) enlarged to 10 μm size in 5 to 10 days. Some cells appeared to recover more readily than others with a more robust surface structure than cells in the panel to the right, which took a longer period of time for recovery. Cells of different dimensions developed from the matrix over the course of 30 days (day 17, left panel). When recovering cells reached a diameter of approximately 10 μm, the overall cell morphology assumed a normal non-dehydrated appearance. Seventeen days later, they were indistinguishable from normal cells (day 17, right). The dehydration-rehydration transformation cycle is summarized in Figure 3B. We repeated this 30-day cycle for several months and observed that cells were able to cycle back and forth between the straw cell and normal morphologies in response to dehydration and rehydration, respectively. However, the number of total viable cells diminished with each successive cycle.

### Chemical compositions of the filaments

**Figure 4:**
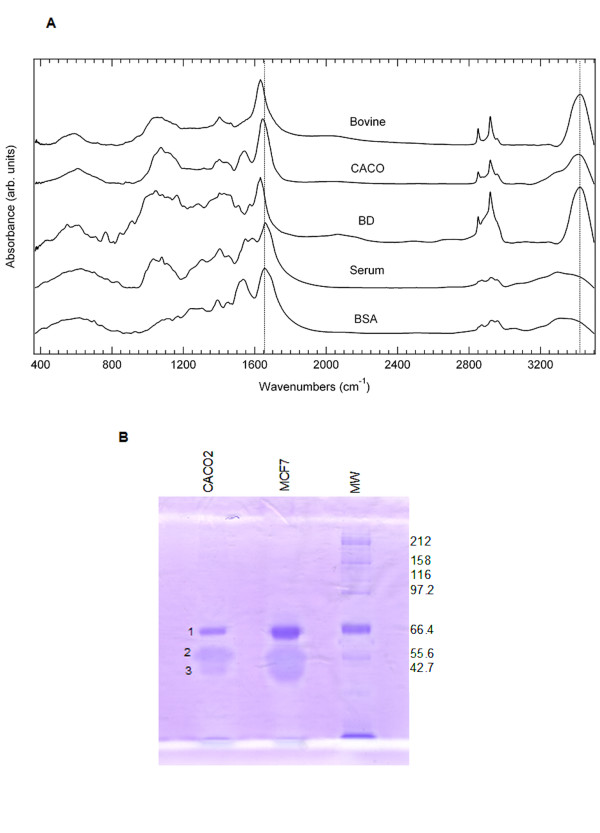
Chemical compositions of the filaments by. **A**. FTIR absorbance spectra of the straw cells (CACO2 and bovine liver), along with proteins (serum, BSA), polysaccharide (blue dextran) controls indicate the straw cells are dominated by bands characteristic of polysaccharide. **B**. SDS/PAGE of the straw cells. Collapsed filaments were dissolved in 1 × SDS loading dye and separated on 8% acrylamide gel. Approximately 1% of loading material was stained positive with Coomasia blue as proteins. Their molecular weights are approximately 50 KD. The In-gel tryptic digestion and de novo peptide sequencing identified band 1 as BSA, which has been reduced by a factor of 5,000 from the starting medium having 10% fetal bovine serum. Band 2 and 3, enriched from straw cells, are likely to be acidic and glycosylated proteins, per their response to Coomasia blue.

Fourier-Transform Infrared Spectroscopy (FTIR) was used to examine the chemical composition of the filaments. Proteins and carbohydrates absorb infrared radiation and the intensity and characteristic bands provide the fingerprint of these molecules [9,10]. Alterations in individual vibrational modes and changes in coupled vibrations can be used to assess hydrogen bonding among proteins and carbohydrates [11,12]. In the filaments, several bands have been shifted relative to the control spectra of proteins (Figure 4A). In the fingerprint region of 900 – 1500 cm^-1^, common bands can be seen between test samples and the controls around 1650 cm^-1^, 1550 cm^-1 ^and 1050 cm^-1^. Bands assigned to hydroxyl stretching modes (3350 cm^-1^) in the protein control samples (spectra labeled Serum and BSA) were decreased in intensity and shifted to higher frequencies (3450 cm^-1^) for peaks in the same range in the test samples, which is possibly due to disrupted hydrogen bonding in the test samples. Amide I band vibrations (carbonyl stretch at 1650 cm^-1^) were shifted to lower frequencies in the test samples, while the CH_2 _stretching modes 2850 cm^-1^) appear sharper in both the absorbance spectra of the straw cells (spectrum labeled CACO) and the blue dextran (spectrum labeled BD), but broader in the spectra of the protein controls. This disparity suggests that the straw cells could contain larger amounts of carbohydrates compared to the controls. These effects mimic those of hydrated trehalose [9]. Taken together, FTIR recorded on CACO2 and bovine straw cells, along with protein (serum, BSA) and polysaccharide (blue dextran) standards indicate that elevated carbohydrate levels most likely contribute to the chemical composition of the straw cells.

Protein composition of the filaments was estimated on SDS/PAGE. Collapsed tube separation on SDS/PAGE showed that approximately 1% (w/w by densitometry) consisted of protein (Figure 4B). Two bands with the molecular weight of approximately 50 kD, along with a BSA band at 66 KD were observed. BSA appeared to be purified by a factor of 5,000 fold when compared to the initial medium having a 10% fetal bovine serum (top band). The protein bands were processed by the in-gel tryptic digestion procedure and micro-sequenced. Among the proteins identified from these two bands using the data dependent acquisition method was a human 40S ribosomal protein (P04643). Western analysis of collapsed straw cells on SDS/PAGE revealed a single band around 212 KD (Data not shown).

Mass spectrometry analysis of partially hydrolyzed filaments revealed a series of six glucose polymers; sulphated glucose polymers and N-acetylglucosamines polymers were among the polymers identified (Figure 5). The polysaccharides were highly acidic and carried multiple negative charges at pH 7.0. The conditions favoring singly charged ions were optimized for detection in both positive and negative ion modes. Three linear polymers differ from each other by a glucose unit (162.06) and a fucose (146.08). A GlcNAC (203.08) monomer was seen in 1 N HCl hydrolyzed bovine liver filaments (Figure 5A). Among identified monomers, fucose (146.08) may be involved in branching. N-acetylglucosamines is a common unit at the reducing end of the bi-antennary structures. Three linear glucose polymers from tubular CACO2 cells differed by a neutral loss of sulphated glucose (242.0) as displayed in Figure 5B. Serial-A sugar ions appear to be the oxygenated form of serial-B sugar ions and serial-C sugar ions were shown to carry two negative charges. These polysaccharides can be summarized in the following structure: R1 and R2 attached to C4 (Figure 5C). The C6 and C2 of the glucose can be present either individually or together and was found in a range of just a few to over a million, when estimated by a gel filtration experiment (data not shown). The acidic makeup of straw cells causes them to be highly hydrophilic. This is the probable reason for their presence in the supernatant of sequential treatment of (1) centrifugation at 16,000 *g*; (2) chloroform: methanol extraction; (3) C18 reverse-phase matrix binding, (4) 100°C, 5 min heat treatment and (5) 5%TCA precipitation.

**Figure 5:**
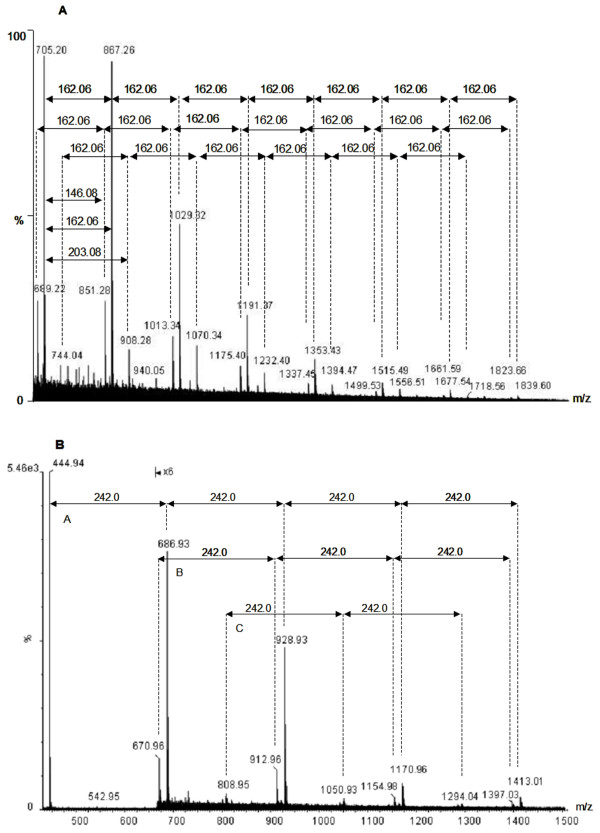
Carbohydrate compositions by Mass spectrometry. **A**. Structural characterization by negative electrospray ionization mass spectrometry. MS analysis has been carried out on oligosaccharides obtained from partial hydrolysis of bovine liver filaments. Spectrum was acquired on Q-Tof micro (Waters) with direct injection through capLC to nanospray ESI in negative ion mode. **B**. Mass spectrum of CACO2 straw cells. Partial hydrolysis with 1N HCl produced linear acidic glucose polymers that differed by neutral loss of sulphated glucose (242.0). Spectra from polysaccharides of those found in urine, such as hyaluronate and dermatan, were acquired as references.

### Radioisotope-labeling

The radioisotope-labeling technique was used to determine if the straw cells were either a product of a metabolically active process involving active cellular growth or if they were a lifeless, physical process involving spontaneous polymerization of cellular materials. Radioisotope labeling of the growth medium using (1-^14^C) glucose determined that the incorporation of ^14^C glucose was more prevalent for dehydrated cells than for normal growing cells, indicating an elevated energy need and increased carbohydrate content of the straw cells (Table 1). More ^14^C was also found in protein fractions from cultures with tubular transformations than from regular growth cultures, indicating that an active amino acid and/or protein metabolism is involved in the process. Overall, the tubular transformation process appeared to be more active metabolically than for normal growth.

**Table 1:** Percent radioactivity recovery in cellular fractions from (1-^14^C) glucose labeled MCF7 cells

	Lipid-phase	Inter-phase	TCA	Ethanol	Aqueous
Zero Incubation	0.8	5	6	0.02	88
Full Incubation	0.3	6	10	0.07	85
Transform ation	0.5 ± 0.2	12 ± 3	21 ± 3	0.4 ± 0.2	66 ± 6

The lipid-phase is the bottom fraction from the chloroform:methanol extraction of the cells. The inter-phase contains cell debris and proteins, and the TCA fraction contains proteins from precipitation of the aqueous fraction. The ethanol fraction contains carbohydrates from the remaining aqueous fraction in 5 volumes of ice-cold ethanol incubated at -20°C over night. The remaining water-soluble fraction contains unused ^14^C-glucose.

### Collapsed filaments found in urine

**Figure 6:**
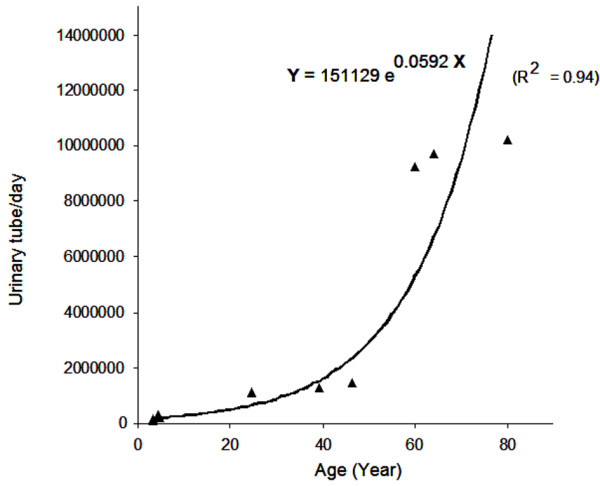
Analysis of urinary straw cells by HPLC. Samples were collected from eight individuals whose urine sugar level was measured using methods adapted from the literature [40, 42]. Quantitative analysis was on C18 reverse phase column chromatography on the 2-min peaks monitored with a refractive index detector. Each data point is the average of multiple injections. The basal level sugar content was selected from urination over a period of 8 to 24 hours.

The HPLC method was used to analyze urinary samples from people at different ages for total carbohydrate quantification. Information on urination volume and time lapse from last urination was collected and used to calculate the carbohydrate production per hour per person, which was in turn converted to number of straw cell structures, based on the equivalency of the carbohydrate mass per tubular structure. Urine carbohydrate levels were found to fluctuate before and after meals, but the basal carbohydrate level remained stable, suggesting that it may be proportional to the straw cell wall material secreted in urine. We discovered that the basal carbohydrate level (lowest urinary carbohydrate content) plotted against age; a polynomial correlation (R^2 ^= 0.94) between tubular cell production and human ages ranged from 3 to 80 years old (Figure 6).

Urinary glycosaminoglycans (GAGs) found widely in animal urine at levels possibly associated with diseases having repeated units of sulphated glucose [13-15], may be derived from these tubular straw cell structures which contain sulphated glucose polymers. Because these filaments were visually detected in urine, the total urinary polysaccharide content may reflect the degree of straw cell formation inside the body, providing an indicator of the aging process.

## Discussion

### Thermodynamic and metabolic rates

Critics have postulated that straw cell structures are the artifacts from dead cells, the polymerization of membrane systems, or growth of protein crystals. Assuming that is the case, then the conversion from a spherical to straw cell is a simple physical deformation process. Therefore, the free energy needed to maintain spherical and tubular structures, built from the same amount of biological material, can be estimated. Following the Helfrich theory [16], the equilibrium shape of a thin membrane covering the vesicle or emulsion droplet is determined by the minimization of the Helmholtz free energy E_H _(or, namely, the "shape energy") of the system

EH=−ΔpV+σA+∫dA[κ2(1R1+1R2−2R0)2+(κ¯R1R2)]
 MathType@MTEF@5@5@+=feaafiart1ev1aaatCvAUfKttLearuWrP9MDH5MBPbIqV92AaeXatLxBI9gBaebbnrfifHhDYfgasaacH8akY=wiFfYdH8Gipec8Eeeu0xXdbba9frFj0=OqFfea0dXdd9vqai=hGuQ8kuc9pgc9s8qqaq=dirpe0xb9q8qiLsFr0=vr0=vr0dc8meaabaqaciaacaGaaeqabaqabeGadaaakeaacqWGfbqrdaWgaaWcbaGaemisaGeabeaakiabg2da9iabgkHiTiabgs5aejabdchaWjabdAfawjabgUcaRGGaciab=n8aZjabdgeabjabgUcaRmaapeaabaGaemizaqMaemyqaeKaei4waS1aaSaaaeaacqaH6oWAaeaacqaIYaGmaaaaleqabeqdcqGHRiI8aOGaeiikaGYaaSaaaeaacqaIXaqmaeaacqWGsbGudaWgaaWcbaGaeGymaedabeaaaaGccqGHRaWkdaWcaaqaaiabigdaXaqaaiabdkfasnaaBaaaleaacqaIYaGmaeqaaaaakiabgkHiTmaalaaabaGaeGOmaidabaGaemOuai1aaSbaaSqaaiabicdaWaqabaaaaOGaeiykaKYaaWbaaSqabeaacqaIYaGmaaGccqGHRaWkcqGGOaakdaWcaaqaaiqb=P7aRzaaraaabaGaemOuai1aaSbaaSqaaiabigdaXaqabaGccqWGsbGudaWgaaWcbaGaeGOmaidabeaaaaGccqGGPaqkcqGGDbqxaaa@5AEA@

The first term in Eq. (1) is given by the pressure difference across the membrane Δp and the volume of the droplet V, while the second term is determined by the interfacial tension σ and surface area A. The integral term in Eq. (1) is often known as the free bending energy, where R_0 _is the spontaneous radius of curvature, κ and κ¯
 MathType@MTEF@5@5@+=feaafiart1ev1aaatCvAUfKttLearuWrP9MDH5MBPbIqV92AaeXatLxBI9gBaebbnrfifHhDYfgasaacH8akY=wiFfYdH8Gipec8Eeeu0xXdbba9frFj0=OqFfea0dXdd9vqai=hGuQ8kuc9pgc9s8qqaq=dirpe0xb9q8qiLsFr0=vr0=vr0dc8meaabaqaciaacaGaaeqabaqabeGadaaakeaaiiGacuWF6oWAgaqeaaaa@2E7D@ is the mean and Gaussian curvature elastic constants, respectively. For a spherical structure, the two primary radii of curvature R_1 _= R_2 _= R, and the first two terms in Eq. (1) are cancelled, so we have

EH−spherical=8πκ(1−RR0)2+4πκ¯,
 MathType@MTEF@5@5@+=feaafiart1ev1aaatCvAUfKttLearuWrP9MDH5MBPbIqV92AaeXatLxBI9gBaebbnrfifHhDYfgasaacH8akY=wiFfYdH8Gipec8Eeeu0xXdbba9frFj0=OqFfea0dXdd9vqai=hGuQ8kuc9pgc9s8qqaq=dirpe0xb9q8qiLsFr0=vr0=vr0dc8meaabaqaciaacaGaaeqabaqabeGadaaakeaacqWGfbqrdaWgaaWcbaGaemisaGKaeyOeI0Iaem4CamNaemiCaaNaemiAaGMaemyzauMaemOCaiNaemyAaKMaem4yamMaemyyaeMaemiBaWgabeaakiabg2da9iabiIda4GGaciab=b8aWjab=P7aRjabcIcaOiabigdaXiabgkHiTmaalaaabaGaemOuaifabaGaemOuai1aaSbaaSqaaiabicdaWaqabaaaaOGaeiykaKYaaWbaaSqabeaacqaIYaGmaaGccqGHRaWkcqaI0aancqWFapaCcuWF6oWAgaqeaGqaciab+XcaSaaa@5039@

For a tubular structure with the inner radius *a*, outer radius *b *and length L, R_2 _= 8, we have

EH−tubular=πσL(a+b)[2−(b−a)2ab]+πκL(1a+1b)+8πκ¯,
 MathType@MTEF@5@5@+=feaafiart1ev1aaatCvAUfKttLearuWrP9MDH5MBPbIqV92AaeXatLxBI9gBamXvP5wqSXMqHnxAJn0BKvguHDwzZbqegyvzYrwyUfgarqqtubsr4rNCHbGeaGqiA8vkIkVAFgIELiFeLkFeLk=iY=Hhbbf9v8qqaqFr0xc9pk0xbba9q8WqFfeaY=biLkVcLq=JHqVepeea0=as0db9vqpepesP0xe9Fve9Fve9GapdbaqaaeGacaGaaiaabeqaamqadiabaaGcbaGaemyrau0aaSbaaSqaaiabdIeaijabgkHiTiabdsha0jabdwha1jabdkgaIjabdwha1jabdYgaSjabdggaHjabdkhaYbqabaGccqGH9aqpiiGacqWFapaCcqWFdpWCcqWGmbatcqGGOaakcqWGHbqycqGHRaWkcqWGIbGycqGGPaqkcqGGBbWwcqaIYaGmcqGHsisldaWcaaqaaiabcIcaOiabdkgaIjabgkHiTiabdggaHjabcMcaPmaaCaaaleqabaGaeGOmaidaaaGcbaGaemyyaeMaemOyaigaaiabc2faDjabgUcaRiab=b8aWjab=P7aRjabdYeamjabcIcaOmaalaaabaGaeGymaedabaGaemyyaegaaiabgUcaRmaalaaabaGaeGymaedabaGaemOyaigaaiabcMcaPiabgUcaRiabiIda4iab=b8aWjqb=P7aRzaaraacdiGaa4hlaaaa@74DD@

**Figure 7:**
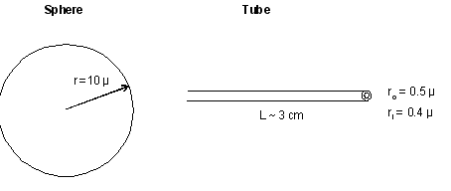
Spherical cell and straw cell dimensions. Two-dimensional view. Measurements were taken from both light and TEM microscopes.

From literature, the κ and κ¯
 MathType@MTEF@5@5@+=feaafiart1ev1aaatCvAUfKttLearuWrP9MDH5MBPbIqV92AaeXatLxBI9gBaebbnrfifHhDYfgasaacH8akY=wiFfYdH8Gipec8Eeeu0xXdbba9frFj0=OqFfea0dXdd9vqai=hGuQ8kuc9pgc9s8qqaq=dirpe0xb9q8qiLsFr0=vr0=vr0dc8meaabaqaciaacaGaaeqabaqabeGadaaakeaaiiGacuWF6oWAgaqeaaaa@2E7D@ of the lipid bilayers are in the range of (0.1–1.0) × 10^-19 ^J (Derek, 2006), as a result, Eq. (2) yields E_H_~10^-18^-10^-17 ^J per spherical cell. These values for carbohydrates, which are the major components of the straw cell wall, are not available. From the TEM measurements, the spherical cell has a typical radius R = 10 μm while the straw cell filaments, has a length of 3 cm, or 3.0 × 10^4 ^μm, an inner radius *a *= 0.3 μm and an outer radius *b *= 0.5 μm (Figure 7). It is immediately noted that the exceptionally large aspect ratio (10^4^) of the straw cell causes the second term in Eq. (3) to dominate any other term in Eq. (2) and (3) containing κ and κ¯
 MathType@MTEF@5@5@+=feaafiart1ev1aaatCvAUfKttLearuWrP9MDH5MBPbIqV92AaeXatLxBI9gBaebbnrfifHhDYfgasaacH8akY=wiFfYdH8Gipec8Eeeu0xXdbba9frFj0=OqFfea0dXdd9vqai=hGuQ8kuc9pgc9s8qqaq=dirpe0xb9q8qiLsFr0=vr0=vr0dc8meaabaqaciaacaGaaeqabaqabeGadaaakeaaiiGacuWF6oWAgaqeaaaa@2E7D@. In addition, the first term in Eq. (3) always contributes accumulatively to the shape energy. Considerable free energy, mainly in the form of the elastic energy, is needed to account for the remarkable spherical-to-tubular shape transformation, with the cell volume increased from 4.2 × 10^3 ^to 2.6 × 10^4 ^(μm^3^) and the cell surface area increased from 1.3 × 10^3 ^to 1.8 × 10^5 ^(μm^2^). We conclude that the tubular structure, as compared to the spherical cell, is heavily disfavored in free energy; which leads us to believe that the spontaneous polymerization of dead cell matrix to tubular structure is an unlikely event.

In reality, the straw cells cannot be simplified to a rod-shaped structure with an overall uniform composition. Not only do they have microscopic hair-like structures covering the surface (Fig.2I), but they also have differences in composition from the tubular center to the filamentous extensions (anisotropic and inhomogeneous) that makes a uniformity assumption untenable. During the spherical to tubular shape transformation, we observed changes in cell volume, surface area, and cell surface composition. The biological transformation we describe differs from a physical event where energy and material exchanges occur throughout the process.

During the reversion of a straw cell to a normal round cell, the tubular center (1 to 2 μm in diameter), houses organelles and grows by enlargement perpendicular to the elongated cell wall (Fig 3A) into a regular round shaped cell. Cellular metabolic rates are determined by the diffusion of ions and biomolecules [17,18] and are expected to be much slower in straw cells than spherical cells because of the narrow cylindrical geometry restricting the intracellular diffusion of solutes compared to a round shaped geometry [19]. The diffusion coefficient of K^+ ^in the t-tubules of skeletal muscle fibers is anomalous and 27% less than its value in free solution [20]. We also observed a much slower growth rate in straw cells (approximately 10 to 15 days) during reversion to regular sized cells, while normal unstressed spherical cells take 2 days to duplicate. It is unclear how straw cells resume a normal round cellular morphology. It is also uncertain as to how ion channels operate during straw cell development.

Straw cell filaments have an outer diameter from 50 to 100 μm (Fig. 1C) and appear to have little cytoplasm (Fig 1D). This is in contrast to fungal apical growth where the elongation of hyphae is typically characterized with a much greater amount of cytoplasm and a distinct endoplasmic reticulum that consists of a network of tubules connected to the nuclear envelope [21,22]. Cell shape in bacteria is often defined by the distinct presence of peptidoglycan, a complex polymer built with glycan chains that are interconnected via peptide crossbridges [23]. Cells with a more complex shape, such as rod-shaped cells, exhibit an additional growth mode responsible for cell elongation [24]. It is unknown if straw cell assembly in regard to the cell wall is similar to bacteria, but glycoproteins and sulphated glucose polymers are primary constituents of the straw cell wall.

Indeed, straw cells can be independently observed in a 5 min experiment. This simply involves collecting any mammalian tissue fluid, placing 1 μL on a glass slide, and observing the droplet under a light microscope. As the droplet dries, the existence of straw cells and their connected filament networks are readily seen [see Additional file 1].

### Proposed model of transformation

We propose that water activity sensors, present on the surface of mammalian cells, survey the hydration status of the environment and may be a type of membrane bound protein kinase(s). In plants, mitogen-activated protein (MAP) kinases [25-27] and phospohlipase D [28] have been upstream responders to the drought stress signaling pathway in Arabidopsis. A study of osmotic-stress-related proteins in rice has identified 12 specific proteins including kinases [29]. Water sensing may be a conserved mechanism throughout eukaryotes and mammalian cells may use similar MAP kinases for intracellular signaling. These sensors, when triggered, invoke a signaling cascade resulting in stress related transcriptional activity in the nucleus. As a result, we postulate that in mammalian cells, one of the potential protective responses involves a dramatic change in cell morphology resulting in the formation of straw cells.

**Figure 8:**
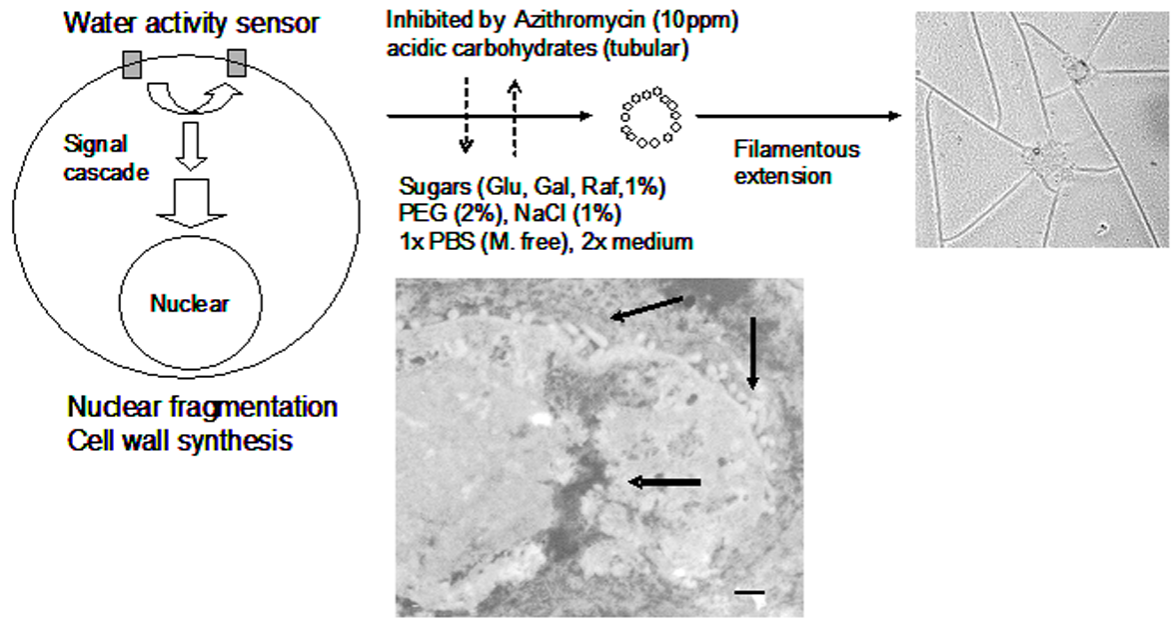
Proposed mechanism of tubular transformation. Water activity sensors on the cell surface survey the hydration status of the environment. Once triggered, they send signals to the nucleus, triggering drastic changes in the entire cell that result in a fragmented nucleus (arrow) and a synthesis of cell wall materials (arrow). The fragmented nucleus, with filamentous branches, resides in the center of fortified tubular structure. Scale bar is 0.2 μm.

Fragmentation of the CACO-2 nucleus (Figure 8, arrow), along with a synthesis of tubular wall was observed during the straw cell transformation process. The time-lapse images were obtained over the entire dehydration process using light microscopy and TEM. These images reveal the presence of polymers assembled along the nuclear membrane (arrows). These subunits are hypothesized to be the building material for the tubular cell wall, presumably, after they have cross-linked to each other.

**Figure 5C:**
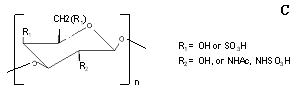
**Carbohydrate compositions by Mass spectrometry.** Structures of identified polysaccharides.

In an attempt to identify the presence of the surface water activity sensors and subsequent proteins involved in the signal cascade, we used small molecules to inhibit straw cell transformation. Initial results indicated that this process could be manipulated and inhibited with naturally occurring compounds as well as synthetic compounds. For example, changes in membrane fluidity by salts and sugars (1%) disrupt the tubular transformation; antibiotics (azithromysin) that inhibit protein synthesis interfered with the tubular transformation by producing filaments that were somewhat truncated.

### Implication in human diseases

The large quantities of straw cells and their ubiquitous nature make them a potential unifying factor across a diversity of age related diseases. The conversion of cells into straw cells may be involved in the initial onset and progression of these diseases, the causes of which are still unknown. Moreover, straw cell formation may be a common physiological response in various types of human degenerative disease. Another area of human health where this phenomenon may play a role is tumor development and proliferation. The hydrophilic, mobile, detachment of straw cells may be a key component of metastasis. As such, hydrophilic straw cells from cancer cells may possibly move around in extracellular fluid and find new locations to grow.

There are many unanswered questions regarding the composition of these tubular cell structures found in a variety of mammalian tissues. For example, what suite of genes encodes the instructions governing the assembly of these tubular structures? What are the necessary molecules for making "unions" and "tees" in the filaments so as to form straw cell networks? Is straw cell transformation a conserved mechanism that mammalian cells use in response to the stress of dehydration and perhaps other stress inducing environmental factors? What is the physiological role of straw cell formation *in vivo*? How much dehydration occurs inside the body either locally or systemically?

Because the cellular transformation is reproducible *in vitro *in a 96-well plate, screening chemical libraries for inhibitory compounds to filamentous transformation is feasible and may result in the ability to control straw cell development *in vivo*. This may have implications in mediating degenerative diseases and tumor proliferation. For example, an *In vitro *assay using azithromysin at 10 ppm resulted in mammalian straw cells with both smaller straw cells and shortened filaments (Wu et al., unpublished data). One type of strategy could include selectively inhibiting protein kinases on the cell surface desensitizing the stress surveillance mechanism.

## Conclusion

Several factors bolster our hypothesis that these tubular structures are indeed special cellular structures that form when a given cell adapts to stress rather than formed from either artifacts or from spontaneous polymerization. These factors include: (1) the pre-existence of tubular structures in all of our examined tissues; (2) the ability to observe filamentous structures via SEM, TEM and light microscopy; the presence of hair-like extensions and small pores or openings on the fixed straw cells (3) the non-production of straw cells in cell-free medium with reagents that changed osmotic potential (salts, PEG, and sugar); (4) the prohibition of spontaneous conversion from sphere-to-straw cells by free energy; (5) the radioisotope labeling of growth medium, showing the transformation to be a metabolically active process; (6) the growth of straw cells into normal cells capable of dividing; (7) the production of truncated straw cells, caused by the interference of antibiotics inhibiting protein synthesis in bacteria; (8) the discovery that tubular walls are made of sulphated glucose polymers after purifying straw cells in gram quantity, and subjecting them to FTIR and Mass spectrometry analysis.

We propose to call these tubular structures transformed through dehydration, "straw cells" because the filaments they produce appear to act as a conduit for the transport of water much like a drinking straw. Similar to the fungal spores, the straw cells are produced by a survival mechanism to protect them from stressful conditions and then revert to a normal morphology when the environment becomes favorable. Understanding the function of these straw cells and developing methods to interrupt their production is may elucidate the cause and effect of the basic pathogenesis in degenerative diseases including malignant tumor metastasis.

## Methods

### Preparation of tubular structures from tissues

Aliquots of 1 μl of extracellular fluid collected from fresh and frozen tissues were placed directly on a glass slide and observed under a light microscope. For quantitative measurements of straw cells, fresh 100 mg bovine tissue slices were harvested in triplicate from the brain frontal lobe cortex, heart muscle, liver, lung and skin tissues and soaked with 100 μL 1 × PBS (buffer: tissue at 1:1, w/w), vortexed briefly and incubated at room temperature for 5 minutes. The resulting slurry was centrifuged at 16,000 *g*, and 2 μL of the supernatant was placed on a glass slide and nitrogen gas dried in a chemical hood for 1 min. The existence of straw cells from tissues was subsequently counted under a light microscope. Incubation of *post mortem *tissues were at room temperature with the lid open. Straw cells were harvested at 24 h intervals using the same soaking procedure as that used for the fresh samples. Frozen normal mouse prostate tissue (Pten+/+; 100 mg) and prostate tumor tissue (Pten-/-; 200 mg) were collected with a laser-capturing micro-dissection microscope. Tissues in microfuge straw cells were stored in liquid nitrogen before use.

### Dehydration and hydration of cultured mammalian cells

CACO2 (colon carcinoma cells, ATCC) at 20,000/ml were cultured in DEME medium supplemented with 20% fetal bovine serum (FBS, ATCC) in culture flasks in a CO_2 _incubator at 37°C. They were transferred to a 4-well chamber (Lab-Tek^®^) with 0.5 ml (1 × volume) culture per well and to a 96-well plate with 50 μl (1 × volume) per well. They were then incubated at 37°C for 2 h and placed at 22°C in either a chemical hood or in a Horizontal Clean Bench (Labconco), again with the lid removed, to start air-dehydration. D1 and adipose D1 cells, MSF7 and THP-1 cells were grown in PMI medium supplemented with 10% FBS (fetal bovine serum). Air-drying was carried out in intervals as well as in a complete dehydration study in which the medium was completely dried off. The drying with the lid removed usually took 8 h in a chemical hood or overnight on the bench. After the cultures were completely dried, transformed cells were isolated from attached cells by a quick rinse with 500 μl 1X PBS, and then centrifugation at 16,000 *g *for 4 minutes. The supernatants containing transformed cells were re-plated with fresh medium to 4-well chambers with 500 transformed cells per well. Dried aliquot was used to count the transformed cells in liquid, and rehydration was carried out at 37°C in a CO_2 _incubator with change of growth medium every two days until newly reemerged cells looked healthy and confluent. Counts of spherical cells against a clear background, as well as 30% Trypan Blue staining, were used to quantify the revival of transformed cells. Subsequent rounds of dehydration rehydration cycle were performed to these cells with a 30-day interval for their recovery in vegetative stage.

### Scanning electron microscopy (SEM) and transmission electron microscopy (TEM)

SEM micrographs were recorded on a Hitachi S-3500 scanning electron microscope (SEM) at 1 to 25 kV on partially air-dehydrated cells in 4-well chambers without any chemical treatment; cells were still submerged in water. The images used background-scattered electrons at magnification ranges from 750× to 2,000×. For TEM, normal cells, transformed straw cells and purified filaments that survived centrifugation at 16,000 *g *were fixed in 2.5% glutaraldehyde, and 3.2% paraformaldehyde in 1 × PBS, pH 7.2 for 1 h, then washed and post-fixed in 1% osmium tetraoxide on ice for 2 h. They were then dehydrated with ethanol at 10 min intervals. Cells were embedded in Embed 812 resin and blocks were cut on a MT2B ultramicrotome, stained in 5% uranyl acetate solution for 15 min and Sato's triple lead for 5 min, and then imaged on H7000 TEM (Hitachi) at 75 kV.

### Actin, nucleic acid and antibody staining

CACO-2 cells were plated onto chamber wells and allowed to adhere to the surface. Cellular dehydration was performed as described above. For fluorescent microscopy, cells were fixed in 4% paraformaldehyde for 10 min at room temperature and then permeabilized in 0.1% TritonX-100 for 2 min. Cells were stained simultaneously with 1:2000 fluorescent phalloidin (Molecular Probes) and 1:2000 SYTOX green (Molecular Probes) for 30 min at room temperature [30,31]. Cells were gently washed once and imaged. For control cells, solutions were prepared in 1 × PBS. To preserve filaments of dehydrated cells, solutions were prepared in 95% ethanol.

Antibody staining of straw cells used rabbit anti-filaments polyclonal IgG as the primary antibody. The antibodies were raised from purified filaments with a small amount of bovine serum proteins. Total IgG was purified from test bleeds using Protein-A bead affinity chromatography (Sigma). The concentration of polyclonal antibody was adjusted to 1 mg/ml and pre-incubated with 1 mg bovine serum protein and then centrifuged to deplete the IgG that recognized the bovine serum proteins. The secondary antibody used was goat anti-rabbit IgG, fluorescent labeled (Alexa Fluor 488, Molecular Probe). Staining of collapsed filaments in solution used a 1:200 dilution of primary antibody incubated for 30 min and a 1:1000 dilution of secondary antibody incubated for 30 min. Negative controls were stained in an absence of either primary antibody or secondary antibody. Staining and cells were imaged in FITC channel and bright field using a fluorescent microscope (Axiovert 200 M, Zeiss) and laser confocal microscope (Zeiss LSM 510) at 10×, 20× and 40×, respectively. Z-stacking and fluorescent lights with various wavelengths were used for visualization. The image was processed with superimposed color to mimic the fluorescent color in particular wavelengths.

### Tube fixation

Freshly prepared straw cells in 4-well chambers were fixed in 0.5 ml 4% paraformaldehyde fixation solution for o/n at 4°C. Next day the fixation solution was removed, and placed in hood for 2 hr to dry. The straw cells were imaged in FITC channel with hundreds of ms exposure time to capture the weak fluorescence. This procedure was used to acquire the images of the hair-like surface structures (Figure 2-I).

### UV radiation

Transformed straw cells were tested for sensitivity to UV-C radiation following procedures in the literature [32,33]. Freshly dehydrated cells were exposed to UV-C radiation in the hood with UV 30 W/G30 T8 (Philips) for 10 min. The dosage was pre-determined by irradiating normal cells in culture for a range of durations, and a 10 min exposure to radiation corresponding to 90% kill was selected for the experiments. Normal and transformed cells before and after dehydration were compared for their survival and revival rates.

### Purification of filamentous extension

Filaments from 4-well-chambered plates were harvested after the removal of most lipids and bovine albumins as described above. The soluble fractions were filtered in a 100 KD spin filter (Microcon, Amicon) multiple times until the pinkish color was depleted. C18 reverse-phase matrix beads (Sep-Pak^®^, Waters) at 1:1 (w/w) were added to the supernatant three times to further remove medium proteins. The supernatants were then extracted three times with chloroform:methanol: water at 2:1:1, the resulting water-phase on the top was precipitated with 5% TCA and centrifuged. The resulting supernatants were then incubated overnight with 80% ethanol at -20°C. The ethanol fractions were centrifuged the next day at -10°C for 10 min at 5,000 RPM in a Benchtop centrifuge (Beckman Coulter) and washed twice with cold, 80% ethanol. Purified filaments were analyzed for their chemical composition by the following physiochemical methods.

### Spectroscopic analysis

FTIR measurements of the filaments were taken using methods from the literature [9,10] The absorbance spectra were obtained using a Bruker IFS 66 v/s spectrometer. The control proteins, polysaccharide standards and test samples were dried extensively (24 hrs in Speedvac, Avanti, 15 mTorr) and placed in a desiccator prior to use. Approximately 3 mg (2–3 weight % of total pellet) were ground to a fine powder with an agate mortar and pestle and mixed with KBr powder. Following this procedure, 100 mg of the resulting powder (weighted to the nearest 0.01 mg) was pressed into 5 mm diameter pellets using a pellet press. The experiments were held under vacuum, excited with a glowbar, and the spectra were collected using a DTGS detector. Background straw cells were collected after evacuation of the sample chamber followed by the sample scan. Absorbance spectra were collected from each of the samples with ranges between 400 – 3200 cm^-1^. Differences in the weights of carbohydrate between disks were normalized on the data station after the infrared spectra were recorded. All spectra were normalized with respect to the strongest recorded peak of 1600 cm^-1^.

The method for carbohydrate composition by mass spectrometry was adapted from the literature [34,35]. Purified filaments were placed at room temperature for days until they collapsed. The water-soluble fraction was hydrolyzed with 1N HCl for 4 h at 70°C for partial hydrolysis and in 6N overnight at 70°C for complete hydrolysis. Electrospray ionization mass spectrometry in both positive and negative ion modes with MS (parent ion) and MS/MS (daughter ion) scans were used to register monomers and polymers of carbohydrates using Q-Tof micro (Waters). Aqueous mobile phase in neutral pH at flow rate of 1 μl/min was directly delivered to the ion source, and the sample cone was set at 3000 volts.

### Radio-labeling

Radiolabeling of cultured cells was done according to published protocols [36,37]. Briefly, one hundredth of one μCi of (1-^14^C) glucose was added to 0.5 ml medium with 10,000 MCF7 cells in a 4-chamber well. The controls were cells in growth medium with zero or full incubation without air-dehydration. The cells were extracted total lipids, proteins, and carbohydrates using sequential organic solvent, trichloroacetic acid, and cold ethanol treatment. 1 ml of moderate ionic cocktail (Ecolite, ICN, Biomedicals) was added to each vial containing the extracted fraction. A multi-purpose scintillation counter (LS 6500, Beckman Coulter) was used to count the radioactivity, using the automatic counting program on both H and C channels. Results from three experiments were averaged to calculate the standard deviations.

### Inhibition Assay

The inhibition assay was developed to identify the inhibitory molecules involved in tubular transformation. Assay compounds, both natural and synthetic, included reverse-phase chromatography fractions from turtle urine and from collapsed bovine liver straw cells, methanol extracts of *fever few *and green tea, sugars, salts, PEGs, and dozens of antibiotics. The analytes, in serial dilutions from 1% to 10 ppb (w/w) were added to wells in 96-well plates (Q-plates 96 'F' well, Genetix, UK) in duplicate, along with 10,000 CACO-2 cells per well. The plates were dehydrated for 18 h with the lid removed in a chemical hood. Activities of the testing molecules were visually assessed in each well to determine the absence of tubular structures on light microscopy.

### Carbohydrate analysis by HPLC

Urine sugar levels were measured using methods adapted from the literature [38-40]. Briefly, 0.5-ml aliquots of urine in a microfuge tube was extracted twice with 1 ml chloroform:methanol (50% v/v) and centrifuged to remove lipids and proteins. The remaining aqueous fraction was adjusted to pH 4.0 using 0.1% acetic acid and injected 100 μl into an analytical HPLC (717 autosampler, 1525 binary pumps, Waters) with Breeze software. A reverse-phase column (C18, 5 um, 4.6 × 150 mm, Waters) chromatography, with mobile phases (0.1 M acetic acid in water and 0.1 NH_4_-acetate in acetonitrile) in a gradient of 2%B/min at 1 ml/min flow rate, were run in continuous mode. A refractive index detector (2410, Waters) and a UV 280 nm detector (2487, Waters) were used in the detection of sugars. Area under the peak was integrated using the breeze software. Urine samples from eight individuals were collected in triplicate with information on the urination volume and time lapse from last urination over an 8 to 24 h period. The total free carbohydrate produced per hour per person was converted to a number of straw cells, based on the equivalency of the carbohydrate content per tubular structure.

### Protein sequencing and Western

Micro-protein sequencing used in-gel tryptic digestion procedure from the literature [41]. After electrophoresis, the gels were stained with Coomassie; protein bands were reduced, alkylated and digested with trypsin and Glu-C proteases to generate peptides. The data-dependent acquisition method (DDA, Masslynx^®^, Waters) was used for the *de novo *sequencing of registered peptide ions. Protein identification used SwissProt, Human and Non- Redundant Protein Databases (NCBI).

Straw cells were resolved on SDS/PAGE and electrophoretically transferred to PVDF membrane (Millipore) at 100 V for 1 h. The membrane was blocked in 5% nonfat milk for 1 h and then incubated with the rabbit anti-tube polyclonal antibody for 2 h at 1:500 dilutions in 1 × PBS supplemented with 1% BSA. The membrane was then incubated with the mouse anti-rabbit IgG HRP antibody (Santa Cruz) for 1 h at 1:1000 dilution in PBS supplemented with 1% BSA. Opti-4CN Substrate and Detection Kits (Bio-Rad) were used for detection of positive bands.

## Authors' contributions

YW conceived and performed most of the studies, RC performed actin staining, DK performed TEM imaging, JH performed SEM imaging, CK prepared cells, JH calculated thermal energies, JR performed FTIR experiment, and DH and JT was involved in sponsoring the project and manuscript preparation. All authors read and approved the final manuscript.

## Supplementary Material

Additional file 1 The pre-existence of straw cells from a tissue. Description: A procedure to observe the tubular structure: (1) collect 1 μl of extracellular fluid from the surface of any frozen bovine liver tissues from a grocery store, (2) place directly on a glass slide and observe the straw cells under a light microscope. As the droplet dries, the existence of tubular structures and their connected networks are revealed. The time-lapse images can be viewed using the Microsoft PowerPoint Presentation slideshow function with a click of mouse for each time point. Click here for file
