# Supplementary material for: Naturally occurring and stress induced tubular structures from mammalian cells, a survival mechanism
Source: BMC Cell Biol. 2007 Aug 16;8:36. doi: 10.1186/1471-2121-8-36 (PMC2000880; doi:10.1186/1471-2121-8-36)
Supplement: Additional file 1 — The pre-existence of straw cells from a tissue. Description: A procedure to observe the tubular structure: (1) collect 1 μl of extracellular fluid from the surface of any frozen bovine liver tissues from a grocery store, (2) place directly on a glass slide and observe the straw cells under a light microscope. As the droplet dries, the existence of tubular structures and their connected networks are revealed. The time-lapse images can be viewed using the Microsoft PowerPoint Presentation slideshow function with a click of mouse for each time point. [file 1471-2121-8-36-S1.ppt]

## Slide 1
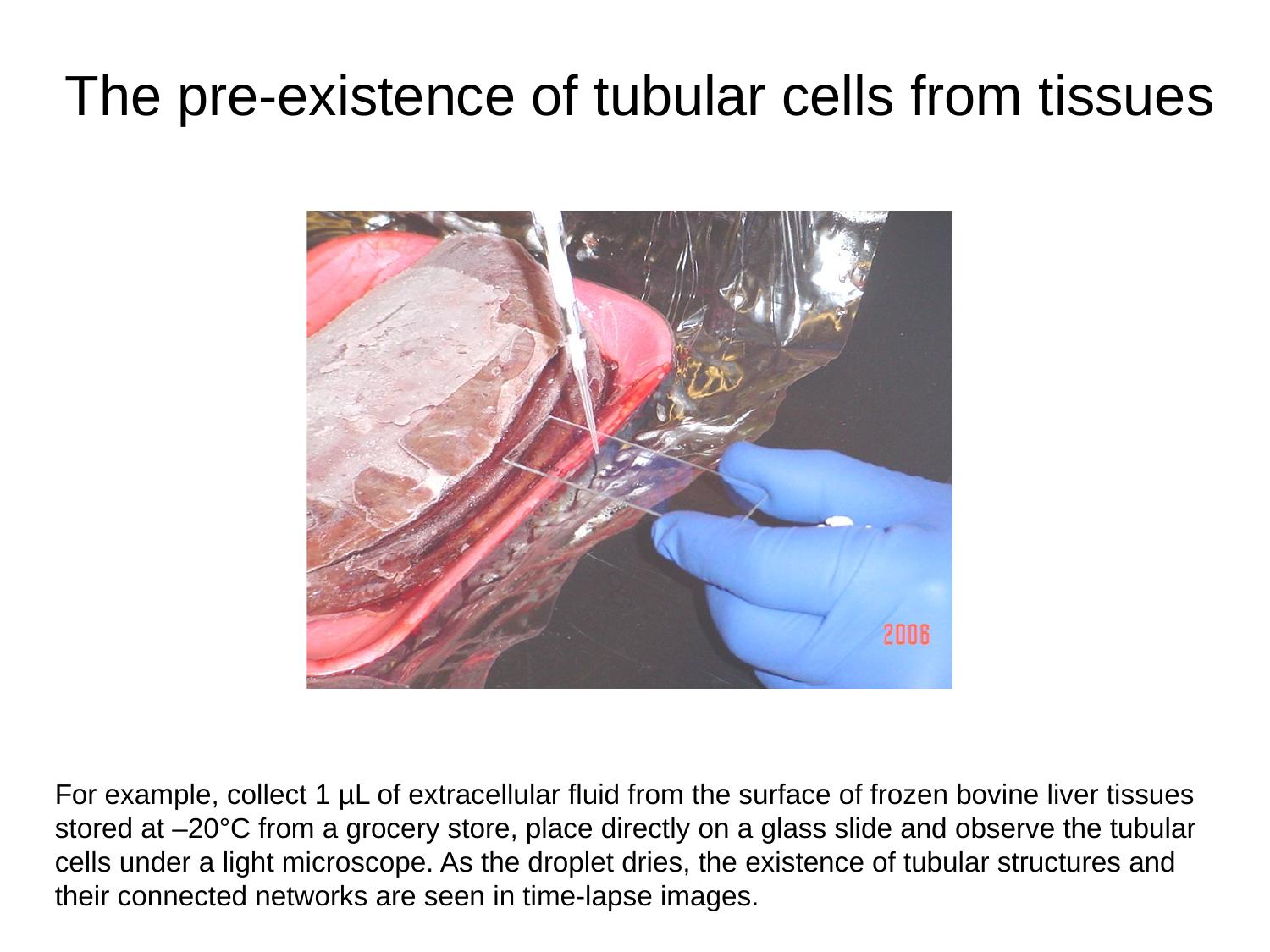

The pre-existence of tubular cells from tissues
For example, collect 1 µL of extracellular fluid from the surface of frozen bovine liver tissues stored at –20°C from a grocery store, place directly on a glass slide and observe the tubular cells under a light microscope. As the droplet dries, the existence of tubular structures and their connected networks are seen in time-lapse images.

## Slide 2
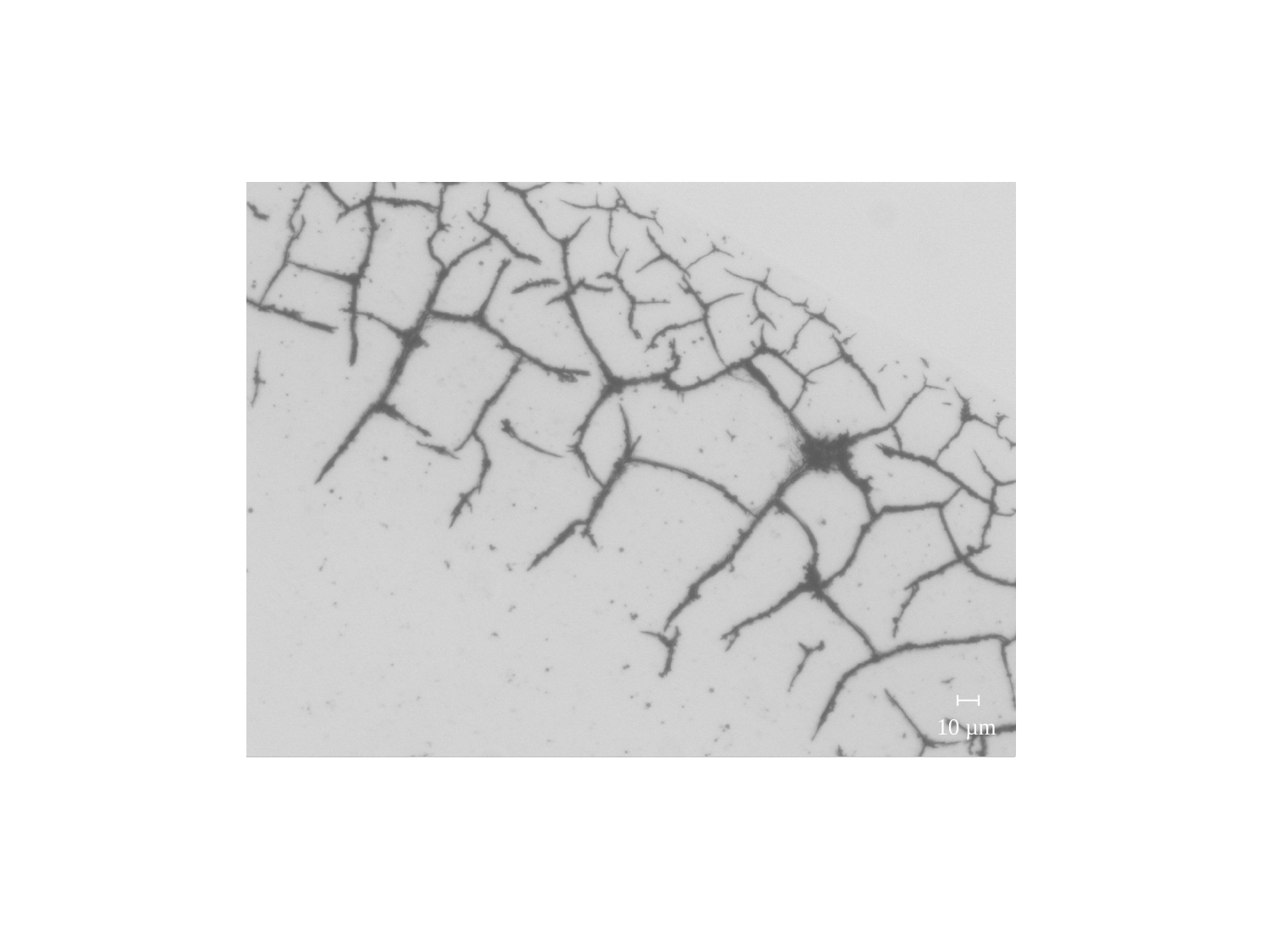

10 µm
10 µm
10 µm
10 µm
10 µm
10 µm
10 µm
10 µm
10 µm
10 µm
